# Zoonotic parapoxviruses detected in symptomatic cattle in Bangladesh

**DOI:** 10.1186/1756-0500-7-816

**Published:** 2014-11-19

**Authors:** Edith Lederman, Salah Uddin Khan, Stephen Luby, Hui Zhao, Zachary Braden, JinXin Gao, Kevin Karem, Inger Damon, Mary Reynolds, Yu Li

**Affiliations:** Epidemic Intelligence Service, Scientific Education and Professional Development Program Office, US Centers for Disease Control and Prevention, Atlanta, GA USA; San Diego State University, Graduate School of Public Health, San Diego, CA USA; International Center Diarrhoeal Disease Research, Dhaka, Bangladesh; Department of Environmental and Global Health, College of Public Health and Health Professions, and Emerging Pathogens Institute, University of Florida, Gainesville, USA; Division of Global Disease Detection and Emergency Response, US Centers for Disease Control and Prevention, Atlanta, USA; Stanford University, Stanford, USA; Poxvirus and Rabies Branch, US Centers for Disease Control and Prevention, Atlanta, USA

**Keywords:** Bangladesh, Bovine papular stomatitis virus, Parapoxvirus, Zoonosis, Pseudocowpox virus

## Abstract

**Background:**

Application of molecular diagnostic methods to the determination of etiology in suspected poxvirus-associated infections of bovines is important both for the diagnosis of the individual case and to form a more complete understanding of patterns of strain occurrence and spread*.* The objective of this study was to identify and characterize bovine-associated zoonotic poxviruses in Bangladesh which are relevant to animal and human health.

**Findings:**

Investigators from the International Center Diarrhoeal Disease Research (icddr,b), the US Centers for Disease Control and Prevention (CDC), and the Bangladesh Department of Livestock Services traveled to three districts in Bangladesh—Siranjganj, Rangpur and Bhola–to collect diagnostic specimens from dairy cattle and buffalo that had symptoms consistent with poxvirus-associated infections. Bovine papular stomatitis virus (BPSV) DNA was obtained from lesion material (teat) and an oral swab collected from an adult cow and calf (respectively) from a dairy production farm in Siranjganj. Pseudocowpox virus (PCPV) DNA signatures were obtained from a scab and oral swab collected from a second dairy cow and her calf from Rangpur.

**Conclusions:**

We report the first detection of zoonotic poxviruses from Bangladesh and show phylogenetic comparisons between the Bangladesh viruses and reference strains based on analyses of the B2L and J6R loci (vaccinia orthologs). Understanding the range and diversity of different species and strains of parapoxvirus will help to spotlight unusual patterns of occurrence that could signal events of significance to the agricultural and public health sectors.

## Findings

### Introduction

Large ruminants have been implicated in the transmission of poxvirus infections to humans for centuries. In India, domestic cattle, buffalo and camels have been observed to harbor incidental infections with zoonotic poxviruses viruses from the *Orthopoxvirus* and/or *Parapoxvirus* genera [1–4]. Though many of these same types of large ruminants are found in neighboring Bangladesh, little is known about the persistence or distribution of zoonotic poxviruses in that country. A single zoonotic outbreak of suspected buffalopox was investigated—and ruled out— in southwest Bangladesh in 1976 [5]. The lack of information pertaining to the identity and burden of zoonotic poxviruses in Bangladesh suggests that both the disease burden in animals and the occupational risks posed by these viruses to animal workers remains largely undefined. The objective of this study was to identify and characterize bovine-associated poxviruses in Bangladesh which are of significance to veterinary public health and which can be transmitted to humans.

In otherwise healthy persons, zoonotic infections with bovine-associated viruses from either the *Orthopoxvirus* or *Parapoxvirus* genus can lead to painful localized pustular lesions or nodules, which generally occur on the upper extremities or face. These lesions are self-limited, resolving slowly over the course of several weeks to months [6]. Arriving at a determination of the precise etiology of a suspected poxvirus-associated infection in either humans or large ruminants necessitates the use of molecular diagnostic techniques, as serology and microscopy do not afford specificity of identification to the level of virus species and the clinical characteristics of these infections are insufficiently distinctive.

In bovines, vaccinia and buffalopox virus infections (orthopoxviruses) can engender high morbidity, with symptoms including malaise, anorexia, and pustular or ulcerated lesions or nodules on the teats and muzzles of adult and juvenile animals, respectively [1, 3, 7, 8]. Symptoms of bovine infection with bovine papular stomatitis virus (BPSV) and pseudocowpox virus (PCPV) (parapoxviruses) can be similar, involving painful erosive papules or vesicles on the muzzle, lips and teats. The exception to this is that BPSV infection in young bovines is sometimes manifest with distinctive ‘horseshoe-shaped’ papular lesions on the hard palate and oral mucosa, which can occur with or without concurrent inflammation of the gingiva [9]. Cryptic and aberrant (sometimes severe) parapoxvirus infections have also been reported in the literature further complicating clinical diagnoses [6, 10–14]. The application of molecular diagnostic methods to the determination of etiology in bovines is important not only for the diagnosis of the individual case, but also to gauge agricultural and human health risks. This by extension allows investigators to form a more complete understanding of patterns of strain occurrence and spread*.*

## Methods

**Figure 1:**
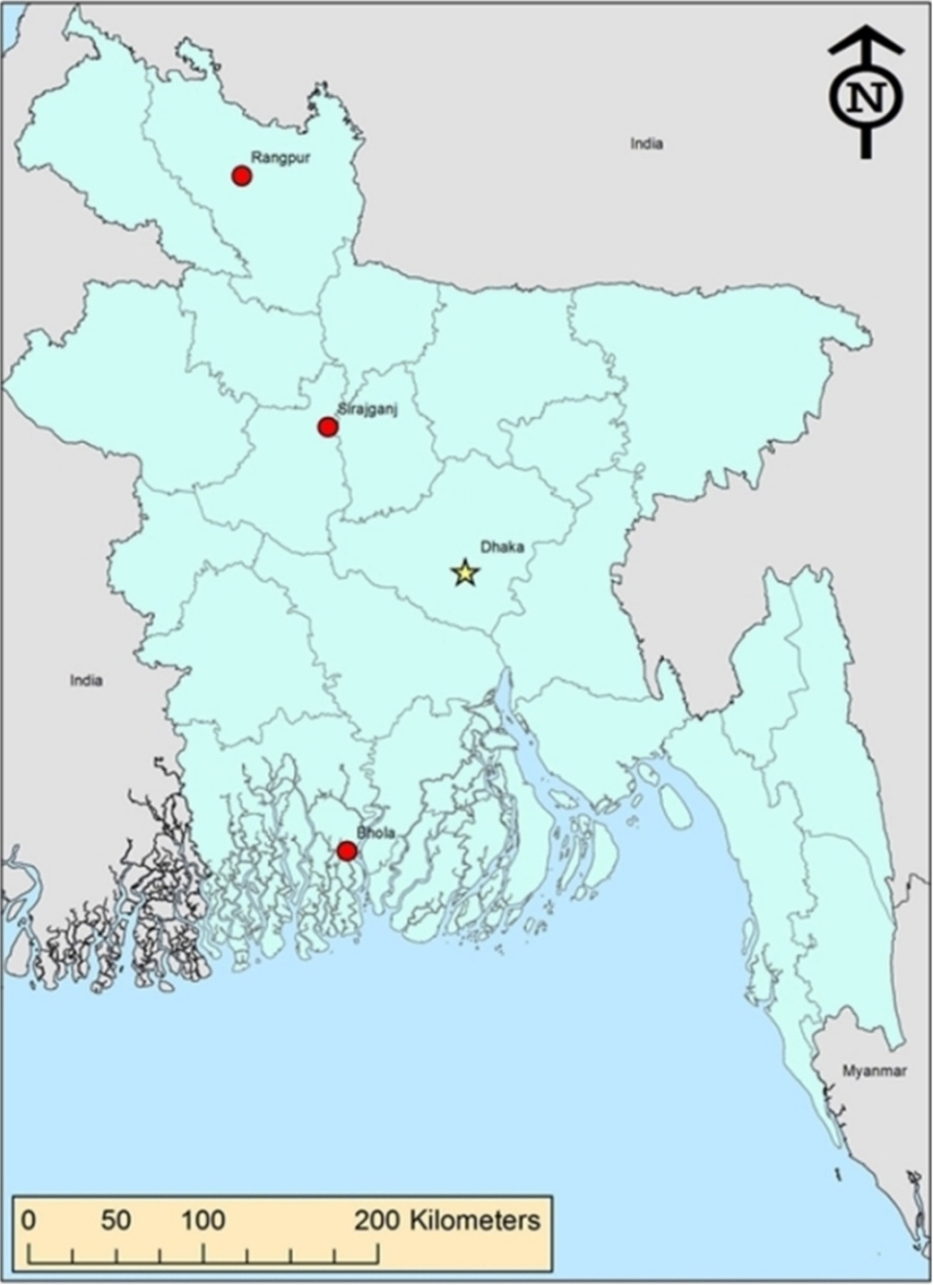
**Map showing investigation sites.** The locations from which specimens were collected during this investigation— Siranjganj and Rangpur within the Rajshahi Division and Bhola in the Khulna Division—are designated on the map.

In April, 2007 investigators from the International Center for Diarrhoeal Disease Research (icddr,b) and the US Centers for Disease Control and Prevention (CDC), along with counterparts from the Bangladesh Department of Livestock services traveled to three locations in Bangladesh—Siranjganj and Rangpur within the Rajshahi Division and Bhola in the Khulna Division (Figure 1)—to collect diagnostic specimens from dairy cattle and buffalo that had symptoms consistent with poxvirus-associated infections. We selected the locations based on recent reports from the Department of Livestock Services of the Bangladesh Government and from a large milk producer cooperative (http://www.milkvita.org) of ‘pox-like’ illnesses in cattle and buffalo. Specimens were collected by veterinarians employed by the Bangladesh Department of Livestock services as authorized statues governing agricultural sanitation. Animal owners provided verbal consent for the collection of whole blood, oral swabs and/or lesion swab or crust specimens, which were collected from 9 symptomatic cattle from the Sirajganj and Rangpur Divisions and 4 buffalo from Bhola (Table 1). For animals that had evidence of scarring, but no active lesions, only blood was collected. Specimens were collected by veterinarians with consent from the animals’ owners. Blood specimens were assayed for the presence of orthopoxvirus antibodies by ELISA [15] and lesion, oral swab, and lesion crust specimens were examined by quantitative real-time PCR (qPCR) for the presence of genus and species level DNA signatures from zoonotic orthopoxvviruses [16] and parapoxviruses [17]. Two PCR amplicons were generated for sequences analysis. The primers and PCR conditions were described previously [18, 19].

**Table 1:** **Laboratory findings and characteristics of animals from which specimens were collected, Bangladesh, 2007**

Animal number	Type/breed	Age	Sex	Condition	Specimen	qPCR*			Serology OPXV ^‡^
						Parapoxvirus (Genus assay)	BPSV ^†^	PCPV ^†^	
**1**	Cow/Jersey	2 mo.	M	Lesion, oral cavity	Oral swab	pos	pos	--	--
					Serum	--	--	--	neg
**2**	Cow/Frisian	7 yr.	F	Lesion, teat	Teat scabs	pos	pos	--	--
					Serum	--	--	--	neg
**3**	Cow/Frisian	4 yr.	F	Lesion, teat	Teat scabs	neg	--	--	--
					Serum	--	--	--	neg
**4**	Cow/Jersey	1 yr.	F	No lesions evident	Serum	--	--	--	neg
**5**	Cow/Frisian	8 yr.	F	Lesion, teat	Swabs/Scabs	neg	--	--	--
					Serum	--	--	--	--
**6**	Cow/indigenous	4 yr.	F	No lesions evident	Serum	--	--	--	--
**7**	Cow/indigenous	5 yr.	F	Lesion, udder	Scabs	neg	--	--	--
**8**	Cow/indigenous	4 yr.	F	Lesion, udder	Scabs	pos	--	pos	--
					Serum	--	--	--	neg
**9**	Cow/indigenous	1 mo.	M	Lesion, oral cavity, muzzle	Swab	pos	--	pos	--
**10**	Buffalo/Monipuri	8 yr.	F	Lesion, udder	Serum	--	--	--	neg
**11**	Buffalo/Monipuri	8 yr.	F	Lesion, udder	Serum	--	--	--	neg
**12**	Buffalo/Monipuri	13 yr.	F	Scar, udder	Serum	--	--	--	neg
**13**	Buffalo/Monipuri	16 yr.	F	No lesions evident	Serum	--	--	--	neg

*All negative for vaccinia-specific CrmB and Opx-generic E9L (negative >45 CT).

^†^BPXV, bovine papular stomatitis virus; PCPV, pseudocowpox virus.

^‡^Sera were examined for the presence of anti-OPX immunolglobulin by ELISA^12^ at 1:100 and 1:400 dilutions.

-- Testing not performed.

Phylogenetic analyses were performed using the Bayesian analysis software package (v1.75, http://beast.bio.ed.ac.uk), BEAST, BEAUti, and Tracer [20]. The analyses run MCMC chain length of 8,000,000 with an HKY nucleotide rate substitution model, strict molecular settings and sampling of every 1,000 states. A strict clock was used and the position of the root was estimated in the tree. The DNA sequences were aligned using BioEdit (http://www.mbio.ncsu.edu/BioEdit/BioEdit.html) and Clustal alignment programs [21]. Data supporting the results of this article are available in GeneBank; accession numbers are included below. The sequences used in the phylogenetic analysis of the B2L amplicon were selected from available parapoxviruses or other high G + C content poxviruses in genbank, include isolates: PCPV_F05_990C [GenBank: JF773694], PCPV_VR634 [GQ329670], PCPV_IT_1303 [JN171852], PCPV_GE3_07 [KF478804], PCPV_JP_IW2010H [AB921003], PCPV_BR_SV721 [KC896641], ORFV_IND 67_04 [DQ263305], ORFV_FIN_F07_3748S [JN773702], ORFV_CHN_Gansu388 [KC485343], ORFV_NZ2 [U06671], ORFVIA82 [AY386263], ORFV_SA00 [AY386264], ORFV_D1701 [HM133903], BPSV_AR02 [AY386265], BPSV_BR_SV819 [JN629089], BPSV_BR_SV716 [KC896639], BPSV_GE_V660 [KF478805], BPSV_JP_IW2010E [AB921002], BPSV_JP_IW2010F [AB921001], SEAV_V842 [AY952943], BPSV_IT_9108 [JN162119], MOCV_T1 [U60315], MOCV_T2_369 [HE977615], SQRV_UK [HE601899]. From the CDC repository, The B2L amplicons were generated from PCPV isolates Bangladesh PCPV_BSH07012, PCPV_BSH07013 (from this study, marked with asterisk), Virginia PCPV_VA0904, and orf isolates ORFV_VA2010910054, BPSV isolates BPSV_VA0982, BPSV_VA09186, BPSV_BSH07005 (GeneBank accession numbers are KF830854, KF830855, KF830856, KF830857, KF830859, KF830860, KF830858, respectively; isolates from this study, marked with asterisk). The sequences used in the phylogenetic analysis of the J6R amplicon include isolates: MOCV_UK [Genebank: JQ269324], MOCV_2008_031 [GQ902057], CROV_Nile [DQ356948], PCPV isolates Bangladesh PCPV_BSH07012 [GQ902051], PCPV_BSH07013 [GQ902052], PCPV_MD06025 [GQ902049], BPSV_BSH07005 [GQ902054], BPSV_WA07058 [GQ902053], PCPV_FIN00_120R [GQ329669], SQRV_UK [HE601899]. From the CDC repository: Virginia PCPV_VA0904J, PCPV_GA08024J, orf isolates ORFV_VA2010910054J, BPSV isolates BPSV_VA0982, BPSV_VA09186J, (GeneBank accession numbers are KF830862, KF830863, KF830861, KF830864, KF830865, respectively).

## Results

**Figure 2:**
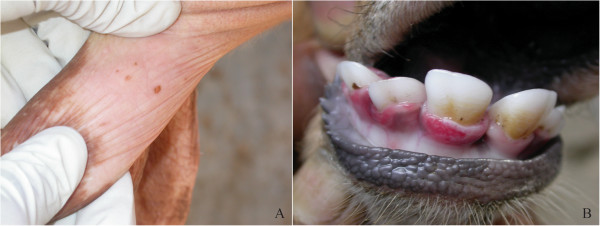
**Photograph of BPSV infection in dairy cattle from Siranjganj.** Panel **(A)** shows parapoxvirus lesion on the teat of animals number 2 described in Table 1. Panel **(B)** shows erosions on the gingiva of animal number 1 from Table 1.

Evidence of BPSV DNA was obtained from lesion material (teat) and an oral swab collected from a single adult cow and calf (respectively) from a dairy production farm in Siranjganj (Table 1, Figure 2). PCPV DNA signatures were obtained from a scab and oral swab collected from a second dairy cow and her calf from Rangpur. No evidence of prior (antibody) or current (virologic) orthopoxvirus infection was detected in any of the 13 animals examined (Table 1).

DNA sequence fragments corresponding to the orthologs of vaccinia B2L and J6R (594 and 630 bp, respectively) were amplified from three of the parapoxvirus-positive specimens described in this study. Primer sets and amplification conditions described elsewhere [18, 19]. The B2L PCR assay has been used frequently for the diagnosis and typing of parapoxviruses, including sealpoxviruses [22]. The pan_pox highGC (J6R) PCR assay has also been frequently employed for the diagnosis and typing of parapoxvirus from clinical samples; one advantage of J6R assay is that it amplify other high G + C content chordopoxvirus, including molluscum contagiousum virus (MOCV) and crocodilepox virus (CROV), and other novel high G + C content poxviruses The J6R assay has been used to amplify an molluscum like poxvirus from an donkey (Fox *et al.* [23]) and two novel poxviruses in central US (Osadebe et al., Emerging Infectious Diseases accepted). The J6R assay is likely to amplify the UK red squirrel poxviruses based on the primer sequences alignment (unpublished results).

**Figure 3:**
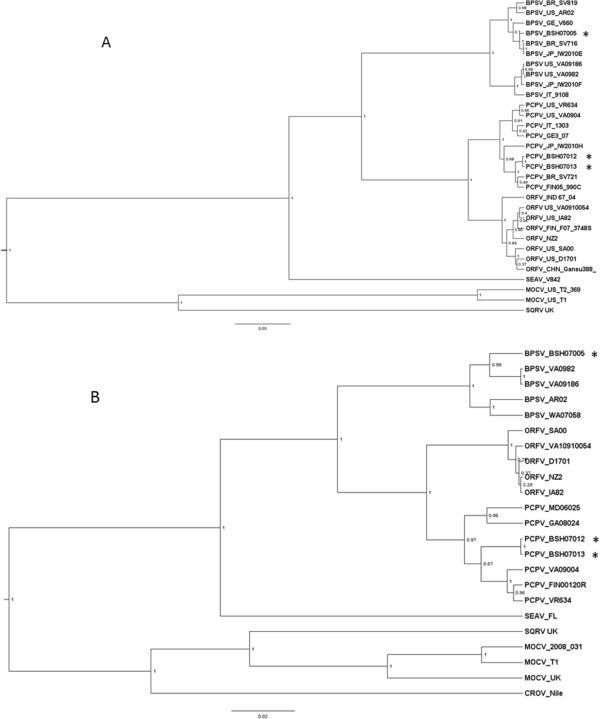
**Phylogenetic trees rendering the relationships between the viruses identified in this study.** The tree is drawn to scale, with branch lengths in the same units as those of the evolutionary distances used to construct the phylogenetic tree. The posterior probabilities were labeled at the each branch with probability values between 0 and 1. **(A)** The phylogenetic trees were constructed from 594 nucleotide of B2L PCR amplicon. **(B)** The phylogenetic tree constructed from 630 nucleotide sequences of J6R PCR amplicons. Sequences from Bangladesh are marked with asterisks.

Phylogenetic trees using both B2L and J6R amplicon DNA sequences were generated to compare the BPSV and PCPV viruses from this study (BPSV_BSH07005, PCPV_BSH07012, PCPV_BSH07013) and other poxviruses to those of other reference viruses and to various viruses from around the world (Figure 3). Panel (A) depicts the phylogenetic tree based on analysis of a 594 bp amplified fragment of the B2L locus [18]; panel (B) the phylogenetic tree based on 630 bp fragment of the RNA polymerase subunit gene J6R [19]. DNA amplicons prepared from viruses in the CDC repository and from sequences derived from Genbank were used for analysis and comparison. Those from the CDC repository include: BPSV_VA9186, _VA0982, ORFV_VA2010910054 [24]; BPSV_WA07058, PCPV_GA08024, PCPV_MD06025 [6]; PCPV_VA0904 [25]. Sequences derived from Genbank are associated with the following strains: PCPV_F05_990C [26]; PCPV_VR634 [27]; BSPB_AR02, ORFVSA00, ORFV_IA82 [28]; BSPB_ITA9108; ORFV_NZ2 [29]; ORFV_D1701 [30].

The B2L tree and J6R tree yield similar topologies at the virus species level for ORFV, PCPV and BPSV: BPSV sequences form a cluster divergent from the PCPV and ORFV clades, while Sealpox virus and MOCV are yet further diverged from those traditional “barn yard” parapoxviruses. Interestingly, Figure 3A shows that PCPV sequence from Bangladesh forms a cluster with PCPV isolates of Brazil (PCPV_BR_SV721) and Finland (PCPV_FIN05_990C), and BPSV sequence from Bangladesh forms a cluster with BPSV isolates from Germany (BPSV_GE_V660), Brazil (BPSV_BR_SV716) and Japan (BPSV_JP_IW2010E), there are no clear geographic linkages for the isolates of both PCPV and BPSV. (This contrasts to the situation for orthopoxviruses, which generally group based on their geographic origins [31].) Individual variations between the two trees may reflect the different levels of sequence conservation between the two loci under consideration. Between closely related sequences PCPV_BSH07012 and PCPV_BSH07013, there is 1 single-nucleotide-polymorphism (SNP) between the B2L amplicons sequences and no SNPs for the J6R amplicon sequences.

## Discussion

In summary, two species of zoonotic parapoxviruses were identified in specimens obtained from symptomatic cattle in Bangladesh. This marks the first occasion that parapoxviruses have been identified in Bangladesh. Unlike capripoxviruses (sheep pox and goat pox), which are endemic to Bangladesh, parapoxviruses can be transmitted to humans [32]. This underscores the importance of determining the infectious etiology of pox-like dermal lesions on domestic bovines to avoid confusion with infections caused by more serious zoonotic pathogens (e.g., buffalopox virus, *Bacillus anthracis*), or with agents that cause severe, notifiable animal diseases (e.g., FMD or Bluetongue) and to ensure that appropriate medical and veterinary interventions are employed. The feasibility of such an approach would be increased in low-resource countries such as Bangladesh by the development of an inexpensive rapid test, such as has been developed for detection of Orthopoxvirus antigen in clinical specimens [33].

People at risk for acquisition of parapoxvirus infections are those whose occupations or food preferences involve direct interaction with infected animals. In most instances human infections are the result of direct contact with infected animals, *via* milking, feeding, or handling of pelts and carcasses [34, 35]. Humans who have occupational exposure to bovines in Bangladesh –through dairy or meat production or other husbandry activities—should take precautions when handling animals with lesions or gingival symptoms. Particular care should be given to attending to wounds on the forearms, hands and fingers as these are the most common sites of parapoxvirus infections in humans [34].

## Conclusions

Aberrant and often serious clinical manifestations of parapoxvirus infections in bovines have been increasingly recognized across the globe [10–13, 36, 37], even as new parapoxviruses are being identified in new large ruminant hosts [25, 38, 39]. Understanding the range and diversity of different species and strains of parapoxvirus will help to spotlight any unusual patterns of occurrence that could signal events of significance to either the agricultural or public health sectors. Insights relevant to the phylogeny and genetic diversity of these viruses may as well impact the design of future animal vaccines. And finally, the findings from this study enable us to hypothesize that zoonotic poxviruses are not uncommon in Bangladesh, however, further studies are needed to determine the prevalence of infection and to identify infection risks for both animals and humans.
